# Diagnostic Value of Serum Biomarkers in Endocrine Dysfunction, Neuronal Injury, and Inflammation Following Traumatic Brain Injury

**DOI:** 10.3390/ijms262110702

**Published:** 2025-11-03

**Authors:** Maria Bouta, Martha Assimakopoulou

**Affiliations:** Department of Anatomy, Histology and Embryology, School of Medicine, University of Patras, Preclinical Medicine Department Building, 1 Asklipiou, 26504 Patras, Greece; mariabouta1@gmail.com

**Keywords:** traumatic brain injury, TBI, hypopituitarism, pituitary dysfunction, neuronal injury, neuroinflammation, serum biomarkers, diagnosis

## Abstract

Traumatic brain injury (TBI) constitutes one of the primary causes of mortality globally. While many survivors fully recover, others experience several chronic complications that, if left untreated, negatively affect the patient’s quality of life. Among these, post-traumatic hypopituitarism (PTHP) represents a common yet poorly recognized condition. The subtle, non-specific nature of pituitary dysfunction symptomatology, its overlap with similar disorders subsequent to TBI, and the lack of sensitive diagnostic tools are the main factors resulting in underdiagnosis of PTHP. The aim of this review is to summarize the existing knowledge, potential clinical utility, and limitations of serum biomarkers that may serve as reliable, minimally invasive tools for assessing pituitary function in the post-TBI period or even predicting late-onset deficiencies. These biomarkers, originating from neuronal damage or the inflammatory response following pituitary injury, can be co-evaluated with basal levels of pituitary and target organs hormones to accurately establish the diagnosis of the condition. Additionally, this review also provides an overview of emerging biomarkers that are currently under investigation and may be incorporated into clinical practice in the future.

## 1. Introduction

Traumatic brain injury (TBI) refers to structural brain lesions caused by external mechanical forces that ultimately lead to dysfunction of the affected area [1]. It has been estimated that TBI annually affects 50 million people around the world [2], with males being typically more susceptible to head trauma [3]. The most common causes of brain injury among patients involve falls (40.5%), collisions (14.3%), and assaults (10.7%) [4], while sports accidents and military-related traumas are gaining particular clinical interest [4,5,6,7]. These mechanical forces result either in blunt or penetrating trauma, including direct brain injury, acceleration–deceleration forces, and blast injury [4,5].

The severity of TBI is determined through several parameters. In particular, the Glasgow Coma Scale (GCS) is currently the gold-standard tool in assessing patient’s eye opening, speech, and motor response in the acute stage of trauma and thus their overall neurological condition [3]. According to this scale, TBI is classified into three categories based on its severity: mild (mTBI, scores 13–15), moderate (scores: 9–12) and severe (scores: 3–8) [4]. Additionally, both the length of loss of consciousness and amnesia following TBI, if present, should be co-evaluated in the final differential diagnosis [4,8,9].

The assessment of TBI severity post-trauma is critical not only for deciding the optimal therapeutic intervention but also for predicting long-term neurological outcomes. If not fatal, head trauma can either resolve over time or cause several acute or chronic complications that, when left untreated, negatively affect the patient’s quality of life and rehabilitation [4,10,11]. Post-concussion syndrome (PCS), chronic traumatic encephalopathy, and pituitary dysfunction are among them [12,13], commonly presenting with symptoms that include fatigue, anxiety, depression, impaired memory, decreased concentration, metabolic disturbances, and behavioral disorders [6,11,12,14,15].

The pituitary gland plays a crucial role in regulating such physical and cognitive functions through the production of central hormones that control the activity of the peripheral endocrine organs. These hormones are secreted either by the anterior lobe (ACTH, TSH, FSH/LH, GH, PRL) or the posterior lobe of the gland (AVP and oxytocin) [16]. Hence, pituitary injury may lead to disruption of one or multiple hormonal axes, resulting in central deficiencies, commonly referred to as post-traumatic hypopituitarism (PTHP) [17]. The symptoms of this condition vary depending on the type of hormone deficiency and may be subtle (mainly when deficits develop in the chronic stage of TBI) or even lethal (e.g., ACTH deficiency causing adrenal crisis), usually occurring within 14 days post-trauma [17,18,19,20]. In most cases, PTHP is transient, with the majority of patients (74–85%) recovering in less than a year post-TBI. Nevertheless, chronic hypopituitarism persisting for over six months, late-onset deficiencies in follow-ups, and dynamic hormonal alterations up to five years following brain injury have also been reported [5,14,19,21,22].

Interestingly, until recent years, PTHP was considered a rare and therefore neglected complication of TBI [11,12]. However, several studies have reported neuroendocrine abnormalities even in mTBI patients, especially those sustaining repetitive mTBIs related to sports or blast exposures [5,6,7,13,18,21]. In a recent systematic review and meta-analysis by Aljboor et al., the pooled prevalence of any axis deficit was estimated at 33%, while deficiencies in multiple axes were present in 7% of patients. Moreover, prevalence tended to be higher within the first three months post-trauma, probably due to physiological adaptive responses to acute illness. Growth hormone deficiency (GHD) was the most common to be found (18%), followed by gonadotropin (16%), ACTH (10%), and thyroid (6%) hormone deficiency [23].

Nonetheless, the prevalence of PTHP differs significantly among studies, which can primarily be attributed to variations in testing methods, participant selection, reference hormonal thresholds, time of sampling, or lack of proper diagnostic tools [14,20,21,24]. At the same time, it is hypothesized that the prevalence of post-TBI pituitary deficiency may actually be underestimated [9]. This is particularly relevant, considering the subtle and non-specific symptoms of this condition, as well as their overlap with acute illness adaptive alterations, medication effects, and other common complications of TBI such as post-traumatic stress disorder (PTSD) [10,15,20,21,22]. Moreover, despite the estimate that around 16.8% of mTBI patients develop PTHP, it remains undiagnosed in most cases, as approximately 50% of all concussions do not receive medical evaluation [6,9,21,25]. These parameters ultimately contribute to delayed diagnosis of hypopituitarism in TBI patients and, thus, poor recovery and neurological outcomes.

Therefore, current scientific research has focused on the search for reliable and easily accessible molecules that could be identified as ideal biomarkers of PTHP in clinical practice. In the following sections, we attempt to review several biomarkers detected in serum that are currently used as diagnostic tools of hypopituitarism, but most importantly, we present the results of recent studies suggesting novel molecules and their prognostic capacity.

## 2. Pathophysiology and Current Diagnostic Tools for PTHP

Numerous studies over the years have attempted to elucidate the pathophysiological sequelae following TBI that ultimately lead to pituitary dysfunction. However, the precise underlying mechanisms are still unclear, with current data supporting that a combination of consecutive events both in the acute and chronic phase of trauma are responsible for this condition [24]. Furthermore, possible differences in mechanisms mediating TBI-induced pituitary deficiency compared to non-traumatic hypopituitarism are a subject of future research [26].

The most widely accepted hypothesis explaining neuroendocrine dysfunction following head trauma involves direct mechanical injury at the time of TBI, especially considering the unique anatomical location of the pituitary gland at the base of the skull. In fact, the gland sits within a depression in the body of the sphenoid bone, the sella turcica, and its posterior lobe is connected to the hypothalamus via a pituitary stalk, which traverses the diaphragma sellae [27,28]. The vulnerability of these structures renders them susceptible to diffuse axonal injury when external mechanical forces are applied. Thus, rapid head acceleration and deceleration in mTBI patients or skull base fractures in more severe cases may cause axonal damage, commonly affecting the pituitary stalk or anterior lobe [1,9,11,19,24,27,29,30].

Nevertheless, primary injury to the pituitary involves not only neuronal but also vascular injury. The hypothalamic–pituitary system is highly distinguished for its complex vascularization, which facilitates the direct regulation of pituitary hormone secretion by the hypothalamus [28]. In particular, each of the two internal carotid arteries gives rise to one superior and one inferior hypophyseal artery. The inferior hypophyseal artery directly supplies the posterior lobe of the pituitary. The superior hypophyseal artery, in addition to directly supplying the pituitary stalk, also provides branches to the median eminence of the hypothalamus. These vessels form a plexus, the hypophyseal portal venous system, which ultimately reaches the anterior lobe by crossing the diaphragma sellae [28,31]. The distinct anatomy of the portal system indicates that it is equally susceptible to injury from external forces, subsequently resulting either in infarction or compressive effects on adjacent structures due to hemorrhage, followed by ischemic insult and necrosis of the gland [1,9,19,20,24,30]. Interestingly, both somatotrophs and gonadotrophs are located in the anterolateral region of the anterior lobe and consequently are supplied by the portal vessels. This observation may provide a plausible explanation for GH and gonadotropin deficiencies being the most common deficits that PTHP patients are diagnosed with [1,11,19,20,31].

The above-described mechanisms of pituitary injury provide a deep insight into the etiology of hormonal abnormalities established in the acute phase of TBI. Yet, studies have reported cases of persistent, deteriorating, or late-onset deficiencies even within five years after the initial head trauma [32]. These data imply an ongoing pathophysiological process in the chronic phase that cannot be attributed to direct mechanical injury [7,19,30,33]. Instead, the primary pituitary damage may trigger a molecular cascade of events at the time of injury that ultimately leads to long-term hypopituitarism. Specifically, neuronal damage-induced glutamatergic excitotoxicity, as well as hypoxia and ischemia resulting from vascular injury, are responsible for further enhancing secondary events already established in the acute phase of trauma, such as blood–brain barrier (BBB) disruption and subsequent neuroinflammation [1]. However, it is worth mentioning that the neurotoxic role of glutamate is highly controversial, especially considering that clinical trials on NMDA antagonists in TBI patients have mostly failed. These findings suggest a potential neuroprotective effect of glutamate, possibly through apoptosis inhibition during the chronic stage of trauma, and imply that its ability to cause neuronal damage is limited within minutes to hours after TBI [34].

**Figure 1:**
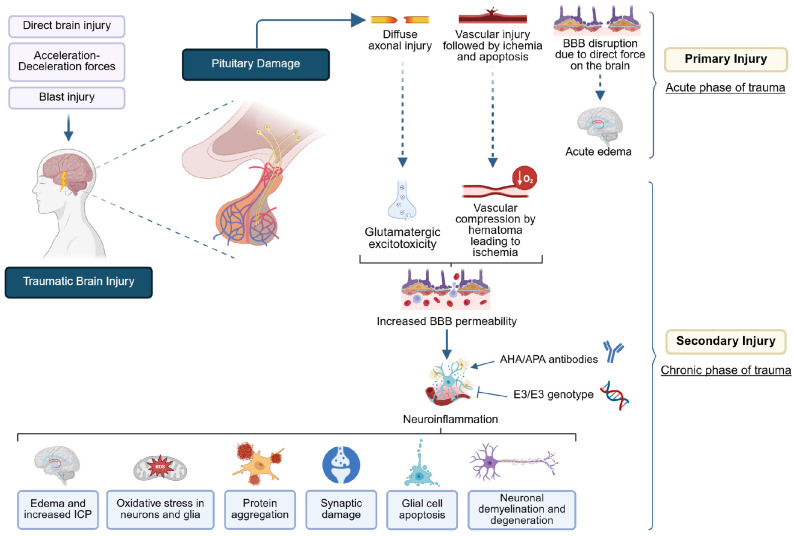
Pathophysiological mechanisms involved in PTHP. Direct mechanical brain injury, acceleration–deceleration forces, and blast injury can cause primary damage to the pituitary in the acute phase of trauma, involving either diffuse axonal injury or vascular rupture followed by ischemia. BBB disruption may also occur at the time of injury due to direct mechanical force, resulting in edema during the acute stage. A cascade of molecular events, commonly referred to as the secondary injury, may be triggered in the chronic phase of trauma, including glutamatergic excitotoxicity and hematoma-induced vascular compression that also contribute to ischemic insult of the pituitary gland. The synergistic effect of these mechanisms contributes to BBB dysfunction and subsequent neuroinflammation. The potential aiding roles of AHA/APA autoantibodies, as well as the inhibitory effect of the E3/E3 genotype on neuroinflammation, are under investigation. The inflammatory response followed by edema formation and blood flow obstruction results in pituitary tissue necrosis through oxidative stress, protein aggregation, synaptic damage, glial cell apoptosis, and neurodegeneration.

Overall, these alterations initiated at the time of TBI due to direct blunt force on the brain and also mediated by the astroglial response to trauma [30] seem to promote acute edema formation followed by increased intracranial pressure (ICP) and, thus, restricted cerebral blood flow if inflammation persists in the chronic stage of TBI [1,9,11,17,19]. The impaired supply of sufficient oxygen to the pituitary gland results in a second ischemic insult and molecular dysfunction of neurons and glial cells, involving oxidative stress, protein aggregation, synaptic damage, and demyelination due to oligodendrocytes’ apoptosis [1,9,19,30]. Figure 1 summarizes the cascade of events that lead to pituitary dysfunction post-TBI. Moreover, a gradually increasing number of studies nowadays highlight the key role of autoimmunity in late-onset hormonal deficits. This hypothesis may also be supported both by brain injury exposing pituitary antigens and increased BBB permeability allowing these antigens to enter the bloodstream and be recognized by the immune system. However, further research is warranted to investigate this association [19,24,35,36].

Considering the complexity of pathophysiological mechanisms mediating pituitary damage, the need for valid diagnostic tools is critical in early recognition and therapeutic intervention if we are to minimize acute or long-term deficiencies. In clinical practice, magnetic resonance imaging (MRI), along with computed tomography (CT), is considered the gold-standard test in the acute stage of TBI [4,25,28]. In particular, CT is a sensitive technique in detecting skull base fractures, while MRI is mainly used for determining the location and type of brain injury, offering 10–20% more precise results compared to CT [3,5,25]. For instance, primary structural damage including vascular and diffuse axonal injury can be detected in the early phase of trauma through MRI in some patients [11]. Moreover, studies have reported MRI-visible alterations in pituitary volume depending on the time elapsed since TBI, with increased pituitary volume observed within the first few days post-TBI, followed by atrophy of the gland or even empty sella in the chronic stage [11,24]. These findings align with the hypothesized ongoing pathophysiology, as enlargement of the gland may indicate edema formation due to underlying neuroinflammation, which over time leads to pituitary necrosis and thus atrophy of the gland. Yet, despite the concordance of the above findings with suspected pathophysiology, imaging is considered to offer limited diagnostic capability and presents minor prognostic value for late PTHP. This is particularly relevant in mTBI patients, in whom structural abnormalities are typically not CT/MRI-visible. Thus, in such cases, normal findings do not exclude late-onset pituitary deficiencies [26]. Furthermore, CT scans are not sensitive to diffuse axonal injury [3], while in another study, MRI was normal for 45% of patients who later developed hypopituitarism [36], indicating that long-term pituitary deficits are primarily associated with microstructural injuries, which are typically less evident on routine imaging compared to visible abnormalities such as stalk rupture or sella fracture. In addition, both methods are unable to detect secondary molecular events, making the prediction of late-onset hypopituitarism challenging [3]. Given the debatable clinical utility of conventional imaging methods among studies, diffusion-weighted imaging (DWI), an advanced technique, has also been proposed for detecting pituitary damage. In particular, DWI is based on the measurement of the apparent diffusion coefficient (ADC), a parameter that reflects changes in the movement of water molecules in tissues. When ischemia occurs, water moves into the intracellular space, resulting in restricted diffusion and thus low ADC, which is depicted as signal hypointensity on the ADC map. Studies have shown decreased ADC in the subacute phase of trauma in the pituitary gland of TBI patients who later developed PTHP. These findings imply that DWI can probably be useful in detecting ischemic insult of the gland, which is not visible on CT or MRI and may ultimately lead to late-onset hormone deficits [11].

Based on this evidence, it can be assumed that radiological findings (including skull base fracture, diffuse brain swelling, and subarachnoid hemorrhage) should mostly be evaluated as risk factors rather than reliable diagnostic criteria for PTHP [5,11,17,20,22,31,33]. Other studies also suggest a positive correlation between TBI severity and long-term hormonal deficits [5,17,19,20,31,37]; however, this hypothesis has recently been doubted given the high prevalence of PTHP in mTBI patients [14,26]. Moreover, the length of hospitalization, as well as the type and cause of injury, are considered additional factors affecting the development of each type of deficit. For instance, it has been reported that ACTH deficiency following TBI is commonly observed in patients involved in traffic accidents [14].

Studies also highlight the genetic component of the pathophysiology of this condition, implying that certain alleles may predict the risk of pituitary deficiency following a TBI. Tanriverdi et al. examined the association between the E3/E3 genotype (coding the APO E3 isoform) and the risk of PTHP in 93 TBI patients and 27 healthy controls. The results showed that the prevalence of hypopituitarism was lower in TBI patients with this genotype (17.7%) compared to those without it (41.9%), suggesting a potential role of APO E3/E3 in predicting favorable outcomes. The implied protective effect of this genotype is hypothesized to be a result of the E3 enzyme’s ability to reduce neuroinflammation triggered by TBI; however, additional research is necessary to clarify the function of the different APO E isoforms post-trauma [38]. A different perspective on genetic predisposition to neuroendocrine abnormalities was provided by Ju et al. In this experiment, including male rats with a history of moderate TBI (F0 generation) and their progeny (F1 generation), it was demonstrated that neurobehavioral abnormalities following TBI may be heritable to offspring regardless of their interaction and social parameters [39]. Notably, the F0 males also presented hypothalamic–pituitary–adrenal (HPA) axis dysfunction, potentially linking endocrine imbalance with inherited stress-related disorders. Nevertheless, there is no sufficient evidence to support this theory in humans as well.

It is therefore clear that no single risk factor is directly indicative of PTHP [14,21]; thus, nowadays, the only reliable method for assessing pituitary function is based upon clinical symptoms and measurement of hormonal levels. Individuals with clinical suspicion of PTHP are referred to an endocrinologist for supplementary diagnostic testing, such as dynamic stimulation tests [25,37]. However, performing dynamic tests on all TBI cases is not feasible, not only because of their considerable cost [31] but also due to the potential risks involved for certain patient groups [11].

## 3. Serum Biomarkers for PTHP

Based on the aforementioned considerations, the need for reliable, minimally invasive methods for diagnosing and predicting the risk of acute or late pituitary dysfunction is currently critical. Specifically, biomarkers found in biofluids of post-TBI patients, such as cerebrospinal fluid (CSF), serum, plasma, and saliva, are attracting increasing scientific interest. Basal levels of such molecules following a TBI could be measured via immunoassay techniques [18] and utilized to determine which patients should be referred for further neuroendocrine testing, and they could even be used to diagnose some types of pituitary deficiency [14,23]. Serum biomarkers, in particular, are preferred as they represent a rapid, easily accessible, and safe method with which to assess a patient’s condition after TBI [25], enhancing the accuracy of PTHP diagnosis, determining trauma severity, and predicting neurological outcomes. Moreover, the detection of basal serum levels of brain-derived biomarkers can contribute to a deeper understanding of the pathophysiology and underlying molecular mechanisms resulting in PTHP, thus leading to targeted therapies and improved rehabilitation [3,40].

### 3.1. Hormonal Biomarkers

The assessment of pituitary deficiency following head trauma is mainly based on the measurement of basal hormonal levels produced by the pituitary and its target organs [29]. Consequently, hormones detected in serum may serve as biomarkers of PTHP.

#### 3.1.1. ACTH Deficiency

HPA axis activation is a crucial event taking place in the acute phase of TBI. Thus, elevated cortisol levels in serum represent a normal adaptive response to trauma within the first hours or days post-injury, with peak cortisol levels detected within 24 h following head trauma. This continuous cortisol secretion, stimulated by ACTH, mediates hemodynamic and metabolic changes while regulating ongoing neuroinflammation [7,19,25]. Elevated cortisol levels in the acute phase suppress inflammation in the central nervous system (CNS). However, when high levels persist in the chronic phase, they are associated with unfavorable outcomes due to neuroinflammation-mediated damage [41].

Central adrenal insufficiency (AI), as a complication of TBI, is the most common pituitary deficiency observed in the acute stage and constitutes a medical emergency due to its life-threatening clinical manifestations (hypotension, hyponatremia, and hypoglycemia). Therefore, immediate intravenous or intramuscular administration of glucocorticoids is essential in patients with suspected AI [24]. Furthermore, hypoadrenalism following TBI is associated with increased mortality of TBI patients as well as poor recovery for chronic patients and long-term pituitary deficiencies in the next 6–12 months [3,12,13,20].

In the acute stage (days 1–7), an ACTH stimulation test is not indicated at least within the first 6 weeks, as it could yield false negative or positive results [5,11,12,26]. According to the British Neurotrauma Group Guidance, the diagnosis should be set through 8:00–9:00 a.m. baseline serum cortisol levels measured in the first 7 days post-TBI only for patients with high risk or presenting signs of hypoadrenalism. Regular testing is not advised due to acute illness [24]. Levels > 15 μg/dL typically rule out AI, while levels < 3 μg/dL are indicative of ACTH deficiency. Cortisol values between 3 and 15 μg/dL require additional testing [11,17,29]. In this case, both serum sodium and glucose could be measured as well (23,31). HPA axis evaluation should be performed in the chronic phase as well (3–6 months), given that 4% of patients develop late onset AI [7,14,18].

However, there are several conditions that could affect basal serum cortisol levels. For instance, in acute illness, severe injury results in changes in corticosteroid-binding globulin (CBG) concentration, making total serum cortisol level a less reliable biomarker of AI [22]. Moreover, etomidate, an anesthetic drug administrated during intubation, can suppress cortisol secretion, leading to decreased cortisol [7,31].

#### 3.1.2. Central Hypothyroidism

Hypothalamic–pituitary–thyroid axis function is assessed during the chronic phase (4–6 weeks or later following a TBI), considering the long half-life of thyroxine [18,24]. Thyroid hormones are considered anabolic hormones; thus, their levels decline during acute illness in order to conserve energy reserves and support essential molecular processes [9,19]. Hence, measuring thyroid hormones values during the acute stage of head injury may result in misinterpretation of hormone levels as pathological due to normal adaptive hormonal changes that occur in response to injury [26].

Central hypothyroidism diagnosis is based on basal serum TSH, free T4 (fT4), and free T3 (fT3) levels. Specifically, low fT4 values (<0.8 ng/dL) [42] combined with decreased or inappropriately normal serum TSH levels (<0.2 mIU/mL) [38] are indicative of thyroid axis dysfunction [22,29]. Decreased fT3 levels (<1.2 pg/mL) [38] are an additional marker of central hypothyroidism, while elevated cholesterol levels are also observed in some studies [23].

Early detection of the deficiency is of critical importance, as fT3/fT4 levels have been correlated with TBI severity and outcome. In particular, fT4 has been associated with mortality [19,23].

#### 3.1.3. Growth Hormone Deficiency (GHD)

As previously discussed, GHD represents the most common pituitary deficiency following head injury, especially in the chronic stage (>3–6 months) [11,24]. GHD is clinically determined via GH stimulation testing, such as insulin tolerance testing (ITT) or preferably glucagon stimulation testing (GST) [11,14], performed 12 months following the injury regardless of trauma severity [21]. Earlier assessment of GHD may be necessary in children, as reported by Gilis-Januszewska et al. [5]. Acute-stage testing is not recommended as it could yield false positive results, since GH is an anabolic hormone and decreasing levels have been reported in over 75% of patients during acute illness [19].

However, performing stimulation testing in all TBI patients would be impossible given the cost and the complexity of dynamic tests [21]. On this basis, Insulin-like growth factor 1 (IGF-1) is currently used in clinical practice as a safe and easily accessible serum biomarker for GHD prognosis in both the acute and chronic stage [2,20,42].

Normal serum IGF-1 values vary by age [33]: 197–476 ng/mL for 18–30 years, 100–494 ng/mL for 31–40 years and 101–303 ng/mL for 41–50 years [38]. Levels lower than 84 ng/mL in the acute phase of TBI could predict GHD at 12 months post-injury [20]. Additionally, IGF-1 is BMI (body mass index)-dependent [14,19]. Decreased IGF-1 levels are typically considered to be predictors of poor cognitive outcome [19], especially in cases of moderate and severe TBI [8], given that both GH and IGF-1 are probably involved in brain repair mechanisms, plasticity, and myelin formation during the post-TBI period [15]. Moreover, low IGF-1 values have also been associated with impaired visual memory [20].

A study by Castellano et al. demonstrated a significant correlation between low serum IGF-1 concentrations and decreased anterior pituitary volume in soldiers who sustained an mTBI [42]. At the same time, in the first study investigating somatotropic axis function in boxers by Kelestimur et al., including 11 active or retired male boxers, GHD was diagnosed in 5 of them (45.4%). When compared to BMI-matched healthy controls, IGF-1 levels were significantly decreased, suggesting that IGF-1 could be indicative of GHD [43]. Tanriverdi et al. pointed out the dynamic hormonal changes still occurring five years post-TBI, reporting similar mean IGF-1 levels in GH-deficient and -sufficient mTBI patients at 12 months post-injury, which significantly dropped in GHD individuals five years later [35].

On the contrary, through a retrospective study, Lithgow et al. observed the absence of correlation between IGF-1 levels and GH status and thus the lack of diagnostic utility of the biomarker. In particular, IGF-1 levels appeared lower in non-GHD than in GHD patients, while all patients except one had normal IGF-1 concentrations regardless of their GH status. The single patient with low IGF-1 had normal values upon GH stimulation testing [8]. Similar results have been observed in multiple recent studies, supporting the hypothesis that low IGF-1 levels might be an indicator but not a reliable biomarker of GHD, while elevated IGF-1 values do not rule out GHD [24,29]. According to Ntali and Tsagarakis, low IGF-1 could establish the diagnosis of GHD only in the presence of other deficiencies [7].

#### 3.1.4. Gonadotropin Deficiency

Gonadotropin (FSH/LH) deficiency is more reliably assessed in the chronic phase of injury, typically within 3–6 months [5,18,24]. Testing shortly after head injury is not recommended, as early alterations in gonadotropin levels may reflect transient stress response rather actual deficiency [31]. For instance, a study showed that 83% and 63% of 101 TBI subjects had decreased LH and FSH levels, respectively, within 10 days post-TBI [19].The diagnosis of gonadotropin deficiency is based on different serum markers for males and females.

In gonadotropin-deficient men, serum FSH/LH levels are low or inappropriately normal, along with reduced total testosterone values (<298 ng/dL) [33]. Approximately 2% of circulating hormone is free, 54% is bound to albumin, and 44% is bound to sex-hormone-binding globulin (SHBG). Thus, total testosterone measurement can be misleading, given that binding proteins’ concentrations can be affected by several factors, such as certain medications [29]. The British Neurotrauma Group Guidance suggests measuring SHBG and albumin levels along with prolactin levels [17,24]. Morning serum levels should be measured on at least two separate days to confirm the diagnosis [19,29]. Decreased FSH/LH and total testosterone values have been associated with trauma severity, while they are considered a sign of poor recovery following a TBI, probably due to the ability of androgens to inhibit cell death. Neuroprotective properties have also been attributed to estradiol [19].

In women, gonadotropin deficiency diagnosis is primarily guided by clinical symptoms including amenorrhea/oligomenorrhea, decreased libido, and fertility disorders [15]. However, hormonal assessment is essential for the differential diagnosis of hypogonadism. In premenopausal women presenting with menstrual irregularities, low or inappropriately normal FSH/LH levels along with low estradiol levels (<27.6 pg/mL) [33] suggest gonadotropin deficiency only after other causes of ovulatory dysfunction, such as thyroid-related abnormalities or hyperprolactinemia, have been ruled out [17,29]. In post-menopausal women, a low FSH concentration is sufficient to support this diagnosis [24].

#### 3.1.5. Hypoprolactinemia and Hyperprolactinemia

During the acute stage of TBI, serum prolactin levels may be elevated, normal or decreased. [7,26]. Hypoprolactinemia, defined by low prolactin levels (<5.0 ng/mL for men and <7.0 ng/mL for women), has been associated with an increased risk of metabolic alterations [29]. Hyperprolactinemia is more commonly reported in the literature and is defined as elevated prolactin levels in serum [38]. Interestingly, it occurs in more than 50% of TBI patients in the acute stage of injury [19]. Increased prolactin concentration may result from the loss of dopamine’s inhibitory action due to pituitary stalk compression caused by TBI. Furthermore, it could reflect a physiological stress response or be induced by antidopaminergic medication [5,31].

Several studies have reported that individuals who sustained severe TBI had higher prolactin levels; thus, a negative correlation between prolactin concentration and GCS is hypothesized [2]. At the same time, prolactin levels measured one day post-injury have been positively correlated with ICP, suggesting a possible prognostic value of the hormone [19]. Lastly, the ability of elevated prolactin to suppress the gonadal axis, inhibiting the secretion of FSH and LH and resulting in hypogonadism, is another important association [31].

#### 3.1.6. Arginine Vasopressin (AVP) Deficiency and Syndrome of Inappropriate ADH Secretion (SIADH)

AVP deficiency or central diabetes insipidus is a condition caused by insufficient AVP secretion due to posterior pituitary dysfunction. Diagnosis is confirmed in the acute stage of TBI by both the clinical features (polyuria and polydipsia) [29] and serum markers, including elevated sodium, creatinine and urea concentrations. Higher levels of plasma glucose are also observed [18,19,26]. AVP deficiency has been linked to increased mortality rate and long-term hypopituitarism in TBI survivors [20,24]. Interestingly, though, some studies suggest that transient AVP deficiency may benefit patients with raised ICP shortly after head injury [24]. In particular, AVP deficiency-induced hypernatremia creates an osmotic gradient between the intracellular and extracellular space, leading to water flux from brain cells to plasma and brain tissue shrinkage due to cellular dehydration. The decreased cerebral volume physiologically reduces ICP, providing a plausible explanation for the clinical observations reported.

Conversely, SIADH is characterized by uncontrolled release of AVP, hence low serum sodium levels are expected [19]. However, the diagnosis can be established only after renal, adrenal, and thyroid dysfunction have been excluded [24].

### 3.2. Neuronal Biomarkers

As previously described, TBI results in neuronal tissue damage and initiates a cascade of multiple secondary events, including elevated ICP. This condition contributes to BBB dysfunction, and thus neuronal and glial cell products are released into circulation. Although such molecules are more abundant in the CSF post-injury, they could be detected in serum and used as biomarkers of brain damage [3,25]. However, their predictive value for specific TBI complications such as hypopituitarism is still under debate. Therefore, their role in PTHP diagnosis is mostly supportive, as they may be co-evaluated with classical endocrine biomarkers and distinguish true pituitary dysfunction from transient, adaptive hormonal responses to trauma or other similar post-TBI syndromes.

#### 3.2.1. Astroglial Cell Damage Proteins

Glial fibrillary acidic protein (GFAP) constitutes a structural component of the astroglial cytoskeleton and is one the two FDA-approved markers of neuronal injury [3,25]. GFAP expression is typically upregulated in the acute stage of TBI, indicating a normal response of astrocytes to brain tissue damage through cytokine and chemokine production and thus the promotion of neuroinflammation [1,30]. Normal serum GFAP levels range between 7 and 20 pg/mL, but post-TBI concentrations can reach up to 69–1196 pg/mL and seem to be correlated with CT findings and TBI severity. Peak levels are detected 20–24 h after injury [3]. Despite the fact that GFAP is exclusively expressed in the CNS, elevated concentrations are detected in non-TBI conditions as well, such as orthopedic injury, stroke, or inflammatory bowel disease, reducing its diagnostic sensitivity to TBI [25]. However, several studies have reported increased serum GFAP levels to be a predictor of poor neurological outcome and mortality in TBI patients 6 months post-injury [40]. In a recent study involving 75 TBI patients, plasma GFAP values were measured at admission and 15 h and 72 h post-injury. Elevated GFAP levels were detected at all time points and were associated with trauma severity, but no correlation with clinical outcomes was found [44]. In another prospective study, Hacioglu et al. investigated the association between GFAP levels and pituitary dysfunction. GFAP concentrations were measured in 40 TBI subjects within the first week and in 22 patients available for follow-up one year after TBI. The results showed no correlation between GFAP levels with hospitalization duration, trauma severity, or pituitary function at both time points, suggesting the inability of the biomarker to predict long-term pituitary dysfunction [3]. Yet, more future studies with larger cohorts are necessary to explore the potential association between the two.

S100 calcium-binding protein B (S100-B) is mainly found in astrocytes, but it can also be detected in the periphery and especially in adipocytes, chondrocytes, and monocytes. It consists of two chains (alpha and beta), with beta chains normally detected in serum at levels ranging from 0.06 to 0.13 μg/dL. However, S100-B concentrations between 0.07 and 0.24 μg/dL following head trauma indicate astroglial damage, with peak levels detected 36 h post-TBI [3,25]. S100-B is gradually attracting more scientific interest due to its potential role as a reliable brain injury biomarker. Several studies have shown that S100-B constitutes a marker of neurovascular damage, while elevated levels, along with increased GFAP levels during admission, are predictors of unfavorable outcomes, especially in patients with severe TBI. Moreover, S100-B’s ability to predict CT scan abnormalities, such as intracranial hemorrhage, has been reported [25,40]. In a prospective study by Shahim et al. on ice hockey players, serum S100-B levels were increased in the acute phase of TBI in 28 concussed players and were correlated with both trauma severity and CT scan results. Moreover, the concentration of the biomarker was associated with symptom duration [13]. In order to clarify if S100-B levels can predict the development of post-concussive complications such as hypopituitarism, Frendl et al. studied a group of 61 mTBI patients 6–12 months after injury. However, no difference in S100-B serum levels was found between patients with (*n* = 10) and without (*n* = 51) pituitary deficiency, thus proving its lack of diagnostic utility for pituitary dysfunction after TBI [45].

#### 3.2.2. Neural Cell Body Damage Proteins

Ubiquitin carboxyl-terminal hydrolase L1 (UCHL-1) is a protein localized in the cytoplasm of neurons normally involved in the degradation of misfolded proteins [3]. It is one of the earliest markers released in both CSF and serum following a TBI, especially among patients who sustain a severe injury [31], with detectable concentrations in biofluids within one hour following the head injury [25]. Despite being typically measured in CSF, several studies have proved the value of UCHL-1 serum levels for predicting TBI neurological outcome and late-onset complications of trauma [3,25]. In particular, serum UCHL-1 levels are reported to have potential in determining TBI severity, especially when combined with GFAP serum levels and CT-visible lesions. Moreover, its levels may be utilized in discriminating between patients with mild to moderate TBI who require neurosurgical intervention and those who do not [25,40]. Babaee et al. observed elevated plasma levels of UCHL-1 as early as 15 h post-TBI; however, no correlation with long-term unfavorable outcomes was found [44].

Neuron-specific enolase (NSE) is a glycolytic enzyme found in the neuronal cytoplasm and, therefore, it cannot be detected in the extracellular space (e.g., biofluids) under normal conditions. However, levels increase within 24 h following a TBI, reaching concentrations above 9 μg/L in adults even after a mild TBI, suggesting its potential as a diagnostic tool when combined with other biomarkers [3]. Nevertheless, NSE’s utility as a reliable clinically used biomarker comes with several limitations. The primary challenge related to the use of this biomarker is its lack of specificity to TBI. NSE is also expressed in red blood cells, meaning elevated levels may make brain injury diagnosis ambiguous if hemolysis, hypovolemic shock due to blood loss, or renal failure coexist [3,40]. Moreover, NSE’s long half-life in blood (exceeding 20 h) renders it an inappropriate marker for evaluating TBI progression over time [3]. Shahim et al. measured serum NSE levels in ice hockey players before and after the end of the playing season. The results showed no significant alterations in NSE concentrations in concussed players, while no correlation with symptom duration was found [13]. Considering the above, the potential diagnostic utility of NSE in clinical practice remains questionable, and more studies need to be conducted to determine the circumstances under which the biomarker can distinguish TBI from other causes that commonly accompany trauma.

#### 3.2.3. Axonal Damage and Demyelination Proteins

The neurofilament light chain (NfL) is a cytoplasmic protein that functions as a structural element of the neuronal cytoskeleton. Normally, NfL is detectable in serum with concentrations ranging from 11 to 17 pg/mL. However, in cases of neuroaxonal damage, such as TBI or neurodegenerative diseases, it is released in the CSF and then, due to BBB dysfunction, into the bloodstream, reaching levels as high as 89–413 pg/mL [3,25]. To date, numerous studies have highlighted the protein’s potential to predict clinical outcomes at 12 months after head injury [3,40]. In particular, elevated NfL serum levels measured at admission have been associated with chronic complications of injury, including degeneration and post-concussion syndrome [40]. Based on these findings, we hypothesize that NfL may also be used as a biomarker of late-onset hypopituitarism, given that this condition is considered a TBI complication that manifests either in the acute or chronic phase. Hacioglu et al. attempted to examine this relationship by measuring serum NfL and hormone levels within both a week and a year post-TBI. During the acute phase, NfL positively correlated with hospitalization duration and cortisol levels, and it negatively correlated with GCS score and IGF-1 levels. These results indicate the marker’s ability to determine TBI severity, verifying the conclusions of previous studies that NfL can be used to discriminate between mTBI and moderate/severe TBI patients. Nevertheless, no association was found between NfL levels and pituitary function one year post-TBI, suggesting that the marker reflects generalized axonal injury rather than damage to a precisely localized anatomical region in the brain [37]. Moreover, elevated NfL levels have been observed in athletes in the absence of TBI, questioning its reliability as a TBI-specific serum biomarker [25].

Tau protein also constitutes a structural component of the neuronal axon. Although it commonly serves as a marker of neurodegeneration, several studies have suggested that brain injury causes hyperphosphorylation of Tau. High levels of phosphorylated Tau tend to aggregate, resulting in neuronal toxicity and microtubule breakdown in the axonal cytoskeleton. In this context, TBI may present tauopathy-like clinical features in some cases [25,30]. In fact, increased levels of serum total Tau (t-Tau) have been reported following a severe or repetitive TBI [40], with a range between 36.44 and 192.34 pg/mL (normal values: 4.48–66.54 pg/mL) [3]. However, the possible role of t-Tau in the diagnosis of TBI remains unclear. It has been suggested that serum t-Tau levels, despite not being representative of the CSF concentrations, can predict unfavorable outcomes in the chronic phase when measured within 24 h post-injury [3,25,40]. Shahim et al. confirmed this hypothesis, reporting elevated plasma levels of t-Tau in concussed ice hockey players. The highest concentrations were detected one hour after injury with a second peak between 12 and 36 h. Interestingly, patients with increased t-Tau in the acute phase presented loss of consciousness or concussion symptoms 10 days after mTBI, suggesting a correlation of the marker’s levels with the recovery time [13]. Conversely, Babaee et al. observed no difference in plasma t-Tau levels between TBI and non-TBI patients, while they failed to find an association with long-term outcomes [44]. Furthermore, the relationship between elevated t-Tau levels and pituitary function needs to be explored, as aggregation of phosphorylated t-Tau in hypothalamic–pituitary axons may result in hypopituitarism. Hacioglu et al. observed a correlation of t-Tau concentrations with GFAP levels in the acute stage of trauma, but no association was found with GCS, hospitalization duration, or pituitary function both in the acute and chronic phase of TBI [37]. These findings confirm the assumption that t-Tau is not only a non-specific biomarker for hypopituitarism prognosis but also has a controversial role in the determination of trauma severity. Additional research is required to clarify its function beyond neurodegenerative diseases (e.g., Alzheimer’s disease), such as in TBI, and its acute or long-term complications, including pituitary dysfunction.

Myelin basic protein (MBP) is a structural protein serving as an essential component of the multilamellar myelin sheath produced by oligodendrocytes in the CNS. Several studies have reported increased levels of this molecule following TBI (especially in mTBI patients and children), with peak levels detected at 48 to 72 h post-injury [3,40]. A combination of direct axonal injury caused by trauma and BBB disruption may explain the elevated serum MBP levels, as myelinated axons are destroyed and the myelin structural elements are released into bloodstream. These findings suggest that MBP could potentially participate in TBI diagnosis, especially in combination with established hormonal biomarkers; however, further studies are expected to validate this hypothesis in the future.

### 3.3. Inflammatory Biomarkers

As noted above, neuroinflammation is not only a physiological consequence of TBI but also a secondary mechanism of injury that may ultimately result in pituitary deficiency. During the initial phase of trauma, neuroinflammation mainly serves to promote brain tissue restoration. However, sustained inflammation over a prolonged period is associated with chronic complications and long-term pituitary deficiency. Neurodegeneration and oxidative stress, caused by the action of inflammatory cytokines, are thought to affect hypothalamic or pituitary brain tissue, leading to dysfunction [1,3,30,41]. In particular, as extensively described in a recent review by Mele et al., head trauma rapidly triggers a physiological process that leads to enhanced BBB permeability. This protective barrier naturally exhibits weak junctions between glial cells in the hypothalamic area, allowing systemic molecules to directly regulate pituitary hormone secretion. However, when TBI occurs, the increased permeability permits peripheral immune cells and inflammatory proteins to infiltrate brain tissue, causing neuroinflammation [1,19].

At the same time, microglial cells, the brain’s innate immune cells, immediately respond to TBI by proliferating at the site of the damaged area and secreting cytokines to organize immune activity. Specifically, polarization of the microglia towards the M1-phenotype results in production of proinflammatory cytokines including IL-1β, IL-6, IFN-γ and TNF-α. In contrast, the M2 microglial activation exerts anti-inflammatory effects through the production of IL-10, typically considered a neuroprotective mechanism with remyelination and regenerative properties. Astrocytes also contribute to neuroinflammation via astrogliosis, a process involving GFAP production, which promotes secretion of cytokines and chemokines. These molecules not only mediate the recruitment of immune cells and edema but also facilitate communication between astrocytes and microglia [1,3,30].

The production of pro-inflammatory cytokines such as IL-1β and IL-18 is mediated by a molecular signaling cascade that is initiated by inflammasome activation in glial cells following a TBI. Numerous studies have observed increased serum concentrations of these molecules in the post-TBI period, while restoration of IL-1β levels has been correlated with optimized rehabilitation [1]. These findings indicate the potential clinical utility of inflammatory factors that leak from CSF into the bloodstream through the disrupted BBB in assessing dynamic alterations in neuroinflammation following TBI. Similar results have been documented for other cytokines as well. For instance, IL-6 serum concentrations can reach as high as 1100 pg/mL (normal: ≤1.8 pg/mL), whereas IL-8 levels may range from 0 to 2400 pg/mL (normal: ≤14.6 pg/mL) following severe head trauma [3]. Increased IL-10 levels have also been reported in the acute phase of TBI, and the measurement of its concentration within 90 min after admission has been shown to differentiate mTBI patients with CT-visible lesions [25].

In addition to soluble inflammatory mediators, circulating cellular biomarkers have also been proposed for the evaluation of neuroinflammation. A study by Elbuken and colleagues highlighted the role of CD34+ progenitor cell count in peripheral blood following TBI in the context of HPA axis dysfunction. In the absence of physiological disruption, an inflammatory response following trauma, such as TBI, promotes activation of the HPA axis and subsequent mobilization of CD34+ cells from bone marrow into circulation, where they contribute to angiogenesis. Results showed CD34+ cell counts to be significantly elevated seven days post-head trauma, supporting their role in tissue repair and vascularization. However no association with basal serum cortisol levels or TBI severity was observed [46]. These findings suggest that although the relationship between CD34+ cells and cortisol levels remains controversial, the interaction of immune and neuroendocrine systems (particularly through HPA activation) warrants more investigation in terms of pituitary dysfunction pathophysiology.

Despite their promising value in clinical practice, inflammatory biomarkers can be interpreted with certain limitations. In particular, the expression of the examined cytokines is upregulated in conditions involving inflammation that are either unrelated to or coexisting with TBI, reducing their diagnostic specificity. However, their co-evaluation with neuronal biomarkers, which as previously described is directly indicative of brain injury, will enhance their utility in neuroinflammation assessment.

Furthermore, communication between the immune and neuroendocrine systems, especially in terms of pituitary dysfunction, is another topic that should be investigated. Santarsieri et al. attempted to explore this correlation by measuring both CSF and morning serum levels of cortisol and inflammatory biomarkers (IL-1β, IL-6, TNF-α) for the first 6 days following TBI. Given the ability of these factors to activate the HPA axis, it was hypothesized that an imbalance between those systems could predict long-term unfavorable neurological outcome. Results showed levels of IL-10 and TNF-α to be higher in serum than CSF, while CSF concentrations of IL-6 were significantly higher than corresponding serum levels in post-injury patients. Moreover, neuroinflammation was highly associated with cortisol levels, indicating that excessive or inadequate concentrations of cortisol (such as in cases of PTHP) may contribute to impaired inflammatory response and chronic complications [41]. These results were also supported by a recent prospective observational study by Lotsios et al. In this study, serum levels of cytokines (IL-6, IL-8, IL-10 and TNF-α), neuronal biomarkers (GFAP and t-Tau), serum cortisol, and concentrations of GCR-α mRNA (which codes the intracellular glucocorticoid receptor) and GILZ mRNA (which codes a downstream protein in the intracellular glucocorticoid cascade) in polymorphonuclear neutrophils were measured both within 48 h and 5–7 days after brain injury (traumatic and non-traumatic). The results showed downregulated expression of GCR-α at both time points. GILZ mRNA levels remained unchanged for the first 48 h, while they were significantly elevated after 5–7 days, indicating upregulated glucocorticoid (GC) signaling. At the same time, IL-6 concentration was increased in the first 48 h compared to 5–7 days post-injury [47]. The negative correlation between GCR-α expression and IL-6 levels with no change in GILZ expression 48 h after head trauma reflects possible GC resistance and thus excess inflammatory response. In contrast, upregulation of GILZ expression along with decreased levels of IL-6 at the second measurement suggests a GC-driven compensatory mechanism targeting neuroinflammation suppression. These findings support the hypothesis that HPA axis dysfunction is highly associated with inflammation following head trauma.

Notably, in a study by Papadimos, it was reported that administration of inhaled nitric oxide (iNO) in patients with TBI and respiratory distress may suppress the activity of the GC receptor in the brain, while promoting its upregulation in peripheral tissues. This mechanism may enhance sensitivity to steroids and thus contribute to the reduction of neuroinflammation [48].

### 3.4. Novel Biomarkers

Although several serum biomarkers have been utilized in clinical practice or proposed over the years, none currently serves as a definitive screening tool for the diagnosis or prognosis of pituitary dysfunction following TBI. Recent studies have attempted to examine potential associations between novel serum markers detected post-injury and long-term hypopituitarism, as well as their possible role in distinguishing this condition from other head trauma-related neurological complications (Table 1).

Among these, anti-hypothalamic antibodies (AHAs) and anti-pituitary antibodies (APAs) have emerged as biomarkers of particular interest. As previously discussed, autoimmunity has been suggested as a possible pathophysiological mechanism of pituitary deficiency following a TBI. In particular, it is hypothesized that either direct or secondary pituitary damage may lead to the exposure of certain pituitary antigens. Concurrently, increased BBB permeability, a normal consequence of TBI, may allow the leakage of such antigens in the systemic circulation and thus result in their recognition by the immune system and the subsequent production of detectable antibodies [19,35,36]. In a prospective study by Tanriverdi et al., serum levels of AHA/APA IgG antibodies were measured in TBI patients three and five years post-injury. AHA and APA were detectable in 60% and 45% of patients, respectively, five years post-trauma, underscoring ongoing dynamic changes in pituitary function during the chronic phase of injury. Notably, these antibodies were absent from serum samples of healthy controls. Moreover, strongly AHA/APA-positive subjects (titers ≥ 1/16) at either time point were more likely to develop late-onset pituitary deficits [35]. In contrast, Vijapur et al. investigated the correlation between persistent hypogonadotropic hypogonadism (PHH) and concentrations of both AHA/APA IgG and IgM isotypes six months post-TBI. The results showed that individuals with PHH had lower AHA/APA IgM levels than healthy controls, implying a possible protective role for IgM isotype against PHH. On the contrary, IgG levels did not differ significantly between the PHH and the non-PHH group [36]. Given that the IgM isotype is the first one to be produced during an immune response and also plays an important role in promoting neuronal regeneration [36], the combined results of these studies suggest that reduced AHA/APA IgM concentrations in the early post-TBI period could lead to pituitary deficits, such as PHH. Conversely, high IgG titers five years after head trauma may be indicative of chronic neuroinflammation and sustained pituitary damage, contributing to long-term dysfunction. These findings validate the complexity of the immunological responses but also the possible distinct functions of both isotypes in late pituitary deficits.

Persistent pathophysiological processes during the late chronic phase have also been documented by multiple other studies from various perspectives. For instance, Taheri et al. investigated the association between circulating microRNAs (miRNAs) and PTHP in 38 patients during the acute phase of trauma (1, 7, and 28 days post-injury) and 25 patients during the chronic phase (5 years post-injury). miRNAs are single-stranded, non-coding RNAs that are 20 to 25 nucleotides in length and are known for their role in the regulation of mRNA transcription and therefore supervision of molecular pathways including proliferation, repair, and apoptosis [49]. Their presence in serum has recently been proposed as a potential biomarker for establishing the diagnosis of several diseases; thus, a possible connection with TBI-induced hypopituitarism needs to be examined as well. Specifically, in each of the measurements taken at all the above time points and upon comparison with blood samples from healthy controls, a statistically significant differential expression of various miRNAs was observed in patients with PTHP. Among those, miR-3610 and miR-126-3p were most consistently identified in individuals with PTHP, while they were absent in those without. In particular, miR-3610 was detected at both the acute and chronic phase of trauma, whereas miR-126-3p was only elevated 5 years after TBI [32]. Liu et al. reported that changes in miRNA levels observed following a TBI may reflect epigenetic alterations in the expression of target genes associated with secondary brain injury [50]. Tufan et al. attempted to explore this association between the development of PTHP and the potential role of miR-126-3p in the pathophysiology of this condition. In the study conducted on mice epigenetically modified with miR-126-3p during the embryonic stage that underwent mTBI, it was concluded that miR-126-3p promotes ACTH secretion, while it inhibits the expression of gonadotropin-releasing hormone (GnRH) in the hypothalamus, resulting in suppression of the hypothalamic–pituitary–gonadal (HPG) axis. Interestingly, though, serum FSL/LH levels were not affected [51]. Based on these findings, we hypothesize that the release of miR-126-3p (and similarly miR-3610) may primarily have a protective effect on neurons during the acute stage of trauma through HPA axis upregulation. However, its prolonged expression could potentially interfere in the normal function of endocrine axes, permanently inhibiting hormone secretion (e.g., GnRH) and leading to long-term pituitary deficits (e.g., gonadotropin deficiency). Overall, miRNAs may hold promise as clinically reliable biomarkers of PTHP, especially given their high sensitivity in peripheral biofluids achieved by miRNA binding to proteins and exosomes [3]. However, more investigation is required in order to identify the target genes that these miRNAs normally regulate, as well as their overall mechanistic role in pituitary dysfunction.

Considering that mTBI is the most prevalent form of head injury and is frequently associated with long-term complications such as pituitary dysfunction, further in-depth analysis for the discovery of serum biomarkers in mTBI patients is currently warranted. It has been reported that an estimated 35–81% of individuals who sustained an mTBI were found to be under the influence of alcohol at the time of trauma. This aspect was investigated in a study by Frendl et al., who examined the potential linkage between serum alcohol markers and prognosis of late-onset pituitary deficits. Concentrations of several biomarkers including S-100, hormones, ethanol (an indicator of alcohol consumption within the last 24 h), and carbohydrate-deficient transferrin (CDT)—which reflects chronic alcohol intake—were measured in serum at admission and 6–12 months post-injury. The results showed similar levels of ethanol in both patients with and without pituitary abnormalities, implying the limited predictive value of ethanol for this complication. However, CDT levels differed significantly between the two groups, with patients with lower CDT concentrations being more likely to develop pituitary deficiency. This observation raises the hypothesis that elevated serum CDT levels (≥1.5%) commonly detected in regular alcohol drinkers may ultimately have a protective effect on pituitary function in the long term. Hence, average alcohol consumption during the month preceding the injury may provide valuable information for the assessment of pituitary function 6–12 months post-injury [45]. Nevertheless, additional studies are needed to confirm these results.

Several mechanisms have been proposed to explain the potential beneficial effects of chronic alcohol consumption. One such mechanism involves the combined action of plasminogen activator inhibitor -1 (PAI-1), a blood pro-coagulant molecule, and alcohol, both of which may contribute to prevent thrombolysis [45]. PAI-1 inhibits tissue plasminogen activator (tPA) and urokinase plasminogen activator (uPA), enzymes responsible for fibrinolysis. Thus, by inhibiting the action of these two molecules, PAI-1 promotes clot formation. As previously mentioned, the unique location of blood vessels supplying the pituitary gland renders them particularly susceptible to trauma. If these vessels are damaged, the physiological hemodynamic processes involved are likely to affect pituitary function. In actuality, PAI-1 has previously been explored as a potential predictor of late-onset pituitary dysfunction by Frendl et al. In this study, various coagulation-related serum markers and pituitary function parameters were assessed both at admission and 6 to 12 months after head trauma in mTBI patients. Among the studied molecules, only PAI-1 was associated with late pituitary dysfunction, with low levels during admission being correlated with late-onset deficits compared to controls. No significant differences were observed between the two groups after 6 to 12 months. Considering the above findings, along with the known role of PAI-1 in coagulation, it may be suggested that sufficient levels of PAI-1 normally suppress tPA activation, which is possibly promoted following TBI. In contrast, decreased concentrations of the pro-coagulant may fail to regulate tPA, resulting in excess fibrinolysis and, ultimately, intracerebral hemorrhage [27]. In this case, damage to the area of the pituitary adjacent to the injured vessel would explain the delayed endocrine deficits. Nevertheless, the role of PAI-1 in TBI and especially pituitary function remains a controversial topic.

## 4. Discussion and Conclusions

Pituitary dysfunction following TBI constitutes an ignored yet not uncommon clinical syndrome, which is associated with increased rates of morbidity and mortality as well as poor quality of life [19]. These complications are particularly common when the diagnosis of PTHP is delayed or completely missed following a head injury. The absence of rapid, accurate, and practical diagnostic tests is regarded as one of the primary factors accountable for limited recognition of this condition, as until now, the identification of hypopituitarism has been based solely on hormonal assessments after symptom onset. Therefore, the discovery of reliable biomarkers that may indicate pituitary injury both in the acute and chronic stage of trauma would be beneficial in clinical practice through the diagnosis of PTHP or, at least, determining patients who require further hormonal evaluation. Moreover, the utility of these molecules would prove to be of substantial significance if they were detected at representative concentrations in easily accessible body fluids, such as serum.

Based on the pathophysiology of pituitary dysfunction leading to central deficiencies, the development of hypopituitarism involves neurological, endocrine, and inflammatory parameters, mostly participating in the secondary cascade of events following trauma [1]. Thus, serum biomarkers can be classified into three categories according to their origin. In addition, a group of promising novel biomarkers is currently under research and is expected to be incorporated into clinical practice within the next few years.

The combined examination of serum biomarkers and risk factors for PTHP will ultimately contribute not only to early recognition of the condition but, most importantly, to the prognosis and prevention of pituitary dysfunction at the initial stages of injury, when trauma complications are still reversible. Moreover, given the non-specific symptoms of hypopituitarism along with their overlap with other similar TBI-related complications, biomarkers may be useful in differentiating the diagnosis between them. For instance, Ramos-Cejudo et al. examined the potential role of corticotropin-releasing factor (CRF) in distinguishing PTSD (a chronic psychiatric syndrome) from typical TBI symptomatology in military veterans. The results showed low CRF serum levels in PTSD patients compared to TBI subjects without the condition, an observation that could plausibly be explained by feedback inhibition of the HPA axis due to chronic stress associated with PTSD [52]. Furthermore, the development of panels including both endocrine and neuronal or inflammatory serum biomarkers could potentially serve as a reliable method of distinguishing true PTHP from transient hormonal alterations as a response to TBI during the acute or subacute phase. For example, decreased levels of fT3/fT4 thyroid hormones along with normal levels of GFAP most likely indicate a physiological adaptive process following head trauma which is aimed at conserving energy and supporting basic cellular metabolism. On the contrary, low fT3/fT4 levels combined with an increased concentration of serum GFAP or IL-8 raise clinical suspicion of underlying structural brain damage and neuroinflammation. In such cases, thyroid function should also be assessed in the chronic stage of TBI to exclude hypothalamic–pituitary–thyroid axis disruption.

Notwithstanding the benefits of using serum biomarkers in the evaluation of pituitary function, there are still several limitations to be taken into consideration before these molecules are included in clinical practice as independent diagnostic tools. To begin with, circulating biomarkers, and especially hormones, are directly influenced by factors not related to pituitary injury itself, such as the age and gender of the patient, BMI and metabolic profile, stress, or time of sampling. It has also been suggested that the type and cause of head trauma may contribute to fluctuations in levels of serum biomarkers between patients. This information indicates that reference values of such markers should be adjusted to biological parameters before being used to assess pituitary function, as they may exhibit variability among TBI subjects. Moreover, interactions between serum biomarkers or between biomarkers and medications are not rare phenomena, which affect their concentrations and lead to misinterpretation of results and diagnostic errors [25,29]. In addition, although biomarkers measured in serum are preferred in terms of safety and easy accessibility, they may not fully reflect actual CSF levels after a TBI. In fact, serum biomarkers are considered to offer limited diagnostic capacity compared to ones detected in CSF. This may be attributed to their lower circulating concentrations, particularly due to their larger volume of distribution [3,25].

Consequently, it is evident that the field of serum biomarkers and their clinical utility in evaluating pituitary function is currently poorly explored. Hence, considering their potential benefits in clinical practice, further research is required to provide a deeper insight into their role during the post-TBI period. The conduct of studies with larger populations—including TBI patients of different severity, ages, types of head trauma, and comorbidities—both in the acute and chronic stage of trauma will allow the derivation of more reliable and representative conclusions regarding the diagnostic and predictive value of each described biomarker. Furthermore, given the inability of most biomarkers to distinguish hypopituitarism from other conditions, the use of panels that combine several non-specific biomarkers with pituitary hormones will not only result in the identification of PTHP but also aid investigation of potential associations among the biomarkers. Of course, co-evaluation of these findings with risk factors and clinical features will enhance the prognostic accuracy of the complication. Lastly, additional research on the underlying mechanisms leading to pituitary dysfunction following trauma is equally important. Exploring the background of the condition will contribute to understanding the pathophysiological cascade of events and discovering new molecules participating in it, which could be tested and used by clinicians in the future.

## Figures and Tables

**Table 1:** Overview of recently studied serum biomarkers: time of detection post-TBI, potential value, and restraints in PTHP diagnosis.

Serum Biomarker	Time of Detection Post-TBI	Diagnostic and Prognostic Value/Benefits	Limitations and Gaps	References
**AHA/APA**	**IgM isotype:** 6 months **IgG isotype:** 3 and 5 years	**IgM isotype:** Possible neuroprotective role in acute phase **IgG isotype:** Potential marker of chronic neuroinflammation	Pituitary and hypothalamic antigens triggering the immune response remain unknown	Tanriverdi et al. [35] and Vijapur et al. [36]
**miR-3610** **miR-126-3p**	Acute phase (1, 7 and 28 days) and chronic phase (5 years) 5 years	- Detected in PTHP patients and absent in controls - High stability in peripheral biofluids improves diagnostic sensitivity	Target genes and role in pathophysiology are still unclear	Taheri et al. [32]
**CDT**	At admission	Elevated levels (≥1.5%) correlated with favorable outcome 6–12 months post-TBI	- Mechanism remains unresolved - Study included only mTBI patients - Differences between social and heavy chronic drinkers not investigated	Frendl et al. [45]
**PAI-1**	At admission	- Elevated levels suggest positive prognosis 6–12 months post-TBI - Reflects vascular response and hemodynamic alterations post-TBI	- Controversy about the role of PAI-1: Protective through preventing hemorrhage, or damaging through coagulation and ischemia - Unclear if PAI-1 participates in adaptive response or pathophysiology	Frendl et al. [27]

## Data Availability

The original contributions presented in this study are included in the article. Further inquiries can be directed to the corresponding author.

## Abbreviations

The following abbreviations are used in this manuscript:

ACTH	Adrenocorticotropic hormone
ADC	Apparent diffusion coefficient
ADH	Antidiuretic hormone
AHA	Anti-hypothalamic antibodies
AI	Adrenal insufficiency
APA	Anti-pituitary antibodies
APO E	Apolipoprotein-E
AVP	Arginine Vasopressin
BBB	Blood–brain barrier
BMI	Body mass index
CBG	Corticosteroid-binding globulin
CDT	Carbohydrate-deficient transferrin
CNS	Central nervous system
CRF	Corticotropin-releasing factor
CSF	Cerebrospinal fluid
CT	Computed tomography
DWI	Diffusion-weighted imaging
FSH	Follicle-stimulating hormone
GC	Glucocorticoids
GCR-α	Glucocorticoid receptor α
GCS	Glasgow Coma Scale
GFAP	Glial fibrillary acidic protein
GH	Growth hormone
GHD	Growth hormone deficiency
GILZ	Glucocorticoid-inducible leucine zipper
GnRH	Gonadotropin-releasing hormone
GST	Glucagon stimulation test
HPA	Hypothalamic–pituitary–adrenal
HPG	Hypothalamic–pituitary–gonadal
ICP	Intracranial pressure
IFN-γ	Interferon gamma
IGF-1	Insulin-like growth factor 1
IL	Interleukin
ITT	Insulin tolerance test
LH	Luteinizing hormone
MBP	Myelin basic protein
MRI	Magnetic resonance imaging
NSE	Neuron-specific enolase
NfL	Neurofilament light chain
PAI-1	Plasminogen activator inhibitor -1
PCS	Post-concussion syndrome
PHH	Persistent hypogonadotropic hypogonadism
PRL	Prolactin
PTHP	Post-traumatic hypopituitarism
PTSD	Post-traumatic stress disorder
S100-B	S100 calcium-binding protein B
SHBG	Sex hormone-binding globulin
SIADH	Syndrome of inappropriate ADH secretion
TBI	Traumatic brain injury
TNF-α	Tumor necrosis factor α
TSH	Thyroid-stimulating hormone
UCHL-1	Ubiquitin carboxyl-terminal hydrolase L1
fT3	Free triiodothyronine
fT4	Free thyroxine
iNO	Inhaled nitric oxide
miRNA	MicroRNA
mTBI	Mild traumatic brain injury
t-Tau	Total Tau
